# Depression Moderates the Effect of Physical Functioning Over Time in Cancer Survivors

**DOI:** 10.1093/geroni/igaa057.479

**Published:** 2020-12-16

**Authors:** Patricia Bamonti, Aanand Naik, Jonathan Bean, Jennifer Moye

**Affiliations:** 1 VA Boston Healthcare System, Brockton, Massachusetts, United States; 2 Center for Innovations in Quality, Effectiveness, and Safety (iQuest), Houston, Texas, United States; 3 Harvard Medical School, Boston, Massachusetts, United States; 4 VA Boston Healthcare, Boston, Massachusetts, United States

## Abstract

Cancer survivors are at-risk for physical functioning (PF) decrements particularly among older adults. However, few studies have examined moderators of PF over time which may guide rehabilitation interventions. This study examined the moderating effect of depressive symptoms on the association between PF at 6 months following cancer diagnosis and PF 18 months after diagnosis controlling for age, education, and treatment status (whether still in treatment). We hypothesized that the association between PF (T1) and PF (T3) would be attenuated by higher depression scores 12 months after diagnosis (T2). Participants (N = 170; Mage 65.3 +/- 9.17, 98.2% male; 81.2% White) with head and neck, esophageal, gastric, or colorectal cancers were recruited from tumor registries at two VAMCs. Self-report measures included demographics, treatment status, depression symptoms (PHQ-9), and physical functioning (PROMIS) were collected. Performance-based measure of PF (SPPB) was administered. Depression symptoms at Time 2 moderated the relation between performance-based PF (SBBP: ΔR2 = .07, F(6, 103) = 10.1, p < .001) but not self-reported PF, PROMIS: ΔR2 = .001, F(6, 110) = 15.3, p =.733. The turning point of non-significance to significance of SBPP T1-T3 was a PHQ-9 score of 7.2. In the absence of depression, the best predictor of future functioning is prior functioning. For those with PHQ-9 scores > 7.2 (28% of the sample), prior functioning does not predict future functioning. Depression should be measured closely with performance-based measures of PF, including gait speed, to improve prediction of future functioning and guide personalized interventions.

